# The Emerging Role of Epigenetic Mechanisms in the Causation of Aberrant MMP Activity during Human Pathologies and the Use of Medicinal Drugs

**DOI:** 10.3390/biom11040578

**Published:** 2021-04-15

**Authors:** Hassan Sarker, Ayman Haimour, Ravneet Toor, Carlos Fernandez-Patron

**Affiliations:** Department of Biochemistry, Faculty of Medicine and Dentistry, University of Alberta, Edmonton, AB T6G 2H7, Canada; hsarker@ualberta.ca (H.S.); ahaimour@ualberta.ca (A.H.); rktoor@ualberta.ca (R.T.)

**Keywords:** MMP, epigenetics, cancer, cardiovascular disease, statins, tetracyclines, bone disease, tuberculosis, COVID-19

## Abstract

Matrix metalloproteinases (MMPs) cleave extracellular matrix proteins, growth factors, cytokines, and receptors to influence organ development, architecture, function, and the systemic and cell-specific responses to diseases and pharmacological drugs. Conversely, many diseases (such as atherosclerosis, arthritis, bacterial infections (tuberculosis), viral infections (COVID-19), and cancer), cholesterol-lowering drugs (such as statins), and tetracycline-class antibiotics (such as doxycycline) alter MMP activity through transcriptional, translational, and post-translational mechanisms. In this review, we summarize evidence that the aforementioned diseases and drugs exert significant epigenetic pressure on genes encoding MMPs, tissue inhibitors of MMPs, and factors that transcriptionally regulate the expression of MMPs. Our understanding of human pathologies associated with alterations in the proteolytic activity of MMPs must consider that these pathologies and their medicinal treatments may impose epigenetic pressure on the expression of MMP genes. Whether the epigenetic mechanisms affecting the activity of MMPs can be therapeutically targeted warrants further research.

## 1. Introduction

Matrix metalloproteinases (MMPs) are a family of at least 23 different Zn^2+^-containing endopeptidases whose abnormal expression contributes to many disorders, including pathological tissue remodeling, arthritis, cancer, and inflammation [1]. MMPs are regulated at the transcriptional, translational, and post-translational levels, as well as by interactions with tissue inhibitors (TIMPs) and acute phase reactants in the circulation (α2-macroglobulin, fibrinogen), which define states of MMP overactivity or underactivity [1,2].

The transcriptional regulation of MMP activity involves transcription factors, DNA polymorphisms, and promoter/enhancer elements, and epigenetic mechanisms, which are the focus of this review. The concept of epigenesis finds its origin in the scientific debate regarding processes that drive development in cells, proposing that chemical reactions involving soluble cellular components can influence cell development [3]. With our increased understanding of chromosomes and DNA, the concept of epigenesis has evolved. Currently, epigenetics builds on the notion that all somatic cells in an organism contain the same DNA, with different cell types exhibiting different patterns of gene expression that can be clonally inherited [3]. Quantifiable epigenetic mechanisms of gene regulation involve chemical modifications on DNA (such as methylation) and DNA-bound histone proteins (such as methylation or acetylation of histone tails) [3].

**Figure 1:**
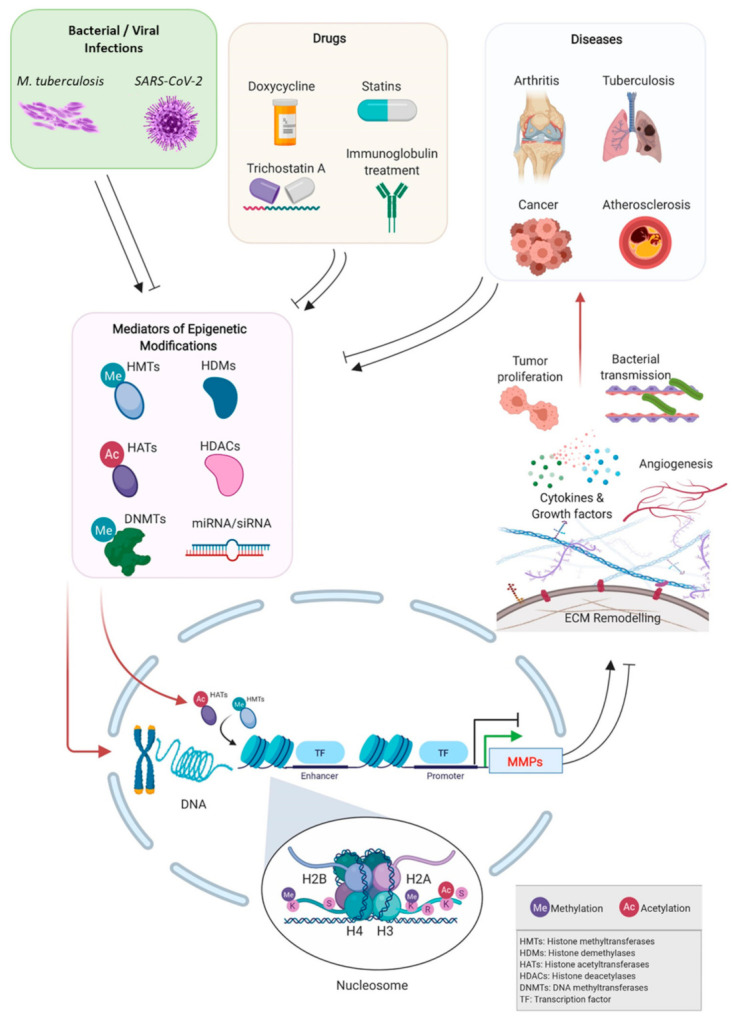
A summary map of the epigenetic regulation of matrix metalloproteinases (MMPs). The epigenetic pressure inducers affecting MMP genes include (i) pathological mechanisms underlying chronic diseases, and (ii) drugs commonly used in treatments for chronic diseases. Epigenetic modifications on DNA, histones, and expression of miRNAs result in dysregulation of the gene expression of MMP, which leads to (i) further promotion of the pathogenesis of an existing disease, and (ii) initiating abnormal remodeling of the extracellular matrix and the regulation of other MMP targets.

Chemical modifications of DNA and histones regulate gene expression by impacting the extent of DNA compaction and therefore the accessibility of transcriptional machinery to genes [4,5]. DNA compaction into chromatin is achieved through the interaction of DNA with an octamer of the core histones [4,5]. This octamer consists of two of each of the histone proteins H2A, H2B, H3, and H4, with approximately 146bp of DNA wrapped around the octamer, forming the nucleosome core particle [4,5] (Figure 1). Multiple nucleosome core particles are connected via linker DNA and the associated linker histone H1 [4,5]. The N-terminal tails of core histones are accessible to post-translational modifications, which can influence gene expression by facilitating or hindering the access of transcription factors and enzymes to the DNA promoter regions [6,7,8] (Figure 1). These modifications include but are not limited to methylation, acetylation, and phosphorylation [9]. In addition to DNA methylation, histone modifications are heritable changes that alter gene expression but not the DNA sequence of genes [9]. Such epigenetic modifications are mediated by DNA methyltransferases, histone acetylases (HATs), histone deacetylases (HDACs), and histone demethylases (HDMs) [9,10,11,12,13] and through the interaction of small non-coding RNA, such as microRNAs and small-interfering RNAs with their corresponding target mRNA, results in gene silencing effects by way of mRNA degradation or transcriptional repression [9,14] (Figure 1). Multiple miRNAs with potential regulatory effects on MMPs have been identified [15], some of which have been confirmed experimentally, which are further discussed here.

Here, we review the current evidence of epigenetic regulation of MMP expression in human diseases including atherosclerosis, arthritis, bacterial infection (tuberculosis), viral infection (COVID-19), and cancer. We also examine evidence of the epigenetic regulation of MMP expression in response to commonly prescribed medicinal drugs with known, but poorly understood, MMP modulatory actions such as statins, which exert epigenetic pressure on many genes and inhibit MMP secretion [16,17,18], and the antibiotic doxycycline, which reduces MMP activity and gene expression [19,20]. The possible involvement of epigenetic mechanisms in the causation of aberrant MMP activity in the settings of acute and chronic diseases and in response to common medicinal drugs is important for predicting disease development and progression, as well as for designing more efficacious therapeutic interventions.

## 2. Epigenetic Regulation of MMPs Expression in Physiological Conditions

Varied gene expression patterns of MMP family members across different cell types may be caused by the cell-type-specific expression of transcription factors and polymorphisms in the promoters of MMP genes, in addition to DNA methylation and histone modifications [21].

The promoter regions of at least fourteen MMP genes (*MMP*-1, −2, −9, −11, −14, −15, −16, −17, −21, −23, −24, −25, −26, and −28) and all four tissue inhibitors of metalloproteinases (TIMP-1, −2, −3 and −4) contain CpG islands that are prone to DNA methylation by DNA methyltransferases [22,23]. DNA methylation generally induces gene repression by preventing transcription factor binding or by attracting methyl-CpG binding proteins that are transcriptional repressive [9,10]. 5-aza-2′-deoxycytidine, a DNA methyltransferase inhibitor, reversed hypermethylation at the *MMP9* promoter (hypermethylation of a promoter represses gene transcription) and concomitantly induced *MMP9* gene expression in lymphoma cell lines [24]. *MMP1* expression is silenced by the methylation of a single CpG site that can be rescued by 5-aza-2′-deoxycytidine [23]. In the colon cancer cell line HCT 116, the deletion of the DNA methyltransferases *Dmt-1* and *Dmt3b* and treatment with demethylating agents induced *MMP3* gene expression without affecting *MMP1* or *MMP2* [25]. Treatment of human non-Hodgkins Raji B lymphoma cells with the demethylating agent 5-aza-2′-deoxycytidine did not induce *MMP3* expression, but instead concentration-dependently increased *MMP10* transcription [25]. These studies suggest that the methylation of CpG sites is a mechanism of MMP gene regulation at the level of transcription, and that DNA methylation/demethylation alters MMP gene transcription in a cell-specific fashion.

Histone-modifying enzymes that introduce chemical modifications on histone tails, particularly H3/H4 acetylation, methylation, or phosphorylation, work alongside chromatin-remodeling complexes to impose control on gene expression by modulating chromatin structures and the accessibility of gene promoters [26,27]. Histone deacetylase 2 and chromatin-remodeling enzyme Mi-2, when recruited to the *MMP9* promoter, reduce H3/H4 acetylation levels and DNA accessibility, consequently repressing *MMP9* gene expression [28]. Similar transcriptional control via histone modifications and chromatin remodeling has been observed for *MMP*-1, −10, and −13 [29,30]. Our understanding of the role played by DNA methylation and histone modifications in regulating the network of MMPs that maintain extracellular matrix homeostasis remains unclear, but it has increased through studies of disease conditions.

## 3. Epigenetic Regulation of MMPs Expression in Pathological Conditions

### 3.1. Cancer

MMPs promote cancer progression through distinct mechanisms, including (i) activation of growth factors [31], (ii) interference with apoptosis pathways [32], (iii) promoting the growth of tumor vasculature [33], and (iv) promoting tissue invasion and metastasis [34]. The last decade has provided mounting evidence that the epigenetic regulation of MMP genes contributes to cancer progression. Strongin et al. demonstrated that the pro-invasive MT1-MMP/MMP-2/TIMP-2 axis is epigenetically regulated in cancer cells [35]. Low migratory breast carcinoma MCF-7 cells exhibited epigenetic transcriptional silencing of MT1-MMP and MMP-2 via the hypermethylation of CpG sites in their respective gene promoters and histone H3 lysine-27-trimethylation. In contrast, highly migratory glioblastoma cells exhibited hypomethylation of CpG sites and low levels of H3 lysine-27-trimethylation [35]. Corroborating these results, DNA methyltransferase inhibition with 5-aza-2′-deoxycytidine increased the transcription of *MT1-MMP* and *MMP2* in pancreatic cancer cells, thereby increasing their invasiveness [36]. In breast cancer cell lines, MCF-7 and MDA-MB-436, 5-aza-2′-deoxycytidine induced the demethylation of CpG sites in the *MMP9* promoter and histone H3 lysine-4-trimethylation (an epigenetic marker of open chromatin-allowing gene transcription) [12], resulting in increased *MMP9* expression [37]. Further, treatment of urothelial cancer cell lines with 5-aza-2′-deoxycytidine or trichostatin A (histone deacetylase inhibitor) induced transcription of 22 MMPs and promoted cancer cell proliferation, migration, and invasion [38]. In glioblastoma cell lines, the expression of microRNAs targeting DNA methyltransferases induced *MMP2* and *MMP9* expression [39]. MMP gene expression can be dysregulated in cancer cells indirectly via epigenetic control of *GRK6*. This gene encodes G protein-coupled receptor kinase 6 (GRK6), an enzyme involved in repressing angiogenesis, tumor growth, and metastasis [40]. GRK6 decreases the expression of the cancer-promoting genes *MMP2* and *MMP7* [41]. In lung adenocarcinoma cells, the *GRK6* promoter is hypermethylated and GRK6 expression is repressed, which promotes cell migration and invasion [41]. Thus, ablation of the GRK6-mediated repression of MMP-2 and MMP-7 may increase the invasiveness of lung adenocarcinoma cells.

Cock-Rada et al. (2012) showed that H3K4 methyltransferase SMYD3 epigenetically regulates the transcription of the *MMP9* gene in a fibrosarcoma cell line (HT1080) and B-cell lymphosarcoma cell line (TBL3) infected with *T. annulata* [42]. Increased *MMP9* mRNA levels in TBL3 cells were associated with greater H3K4me2 and H3K4me3 marks in the regulatory region of the *MMP9* promoter. The elevated histone marks were determined to be the result of SMYD3, as it was the only methyltransferase significantly induced in TBL3 cells. SMYD3 induces transcriptional activation by binding to the 5′-CCCTCC-3′ or 5′-GGAGGG-3′ DNA sequences in the promoter region of genes including *MMP9*. Furthermore, HT1080 cells transfected with siRNAs against SMYD3 resulted in decreased cell migration and proliferation accompanied by decreased levels of *MMP9* mRNA and protein. Similar results were achieved by knocking down only *MMP-9*. Therefore, SMYD3 acts as an epigenetic regulator of *MMP9*, and consequently affects cancer invasion.

*MMP9* has also been reported to be the target of miR-211 [43]. In multiformed grade IV glioblastoma specimens, miR-211 expression is suppressed as a result of abnormal methylation of its promoter [43]. An inverse relationship between the expression of miR-211 and MMP-9 protein levels was observed. Asuthkar et al. (2012) also showed that miR-211 overexpression and shRNA specific for *MMP9* (pM) treatments was associated with anti-proliferative and apoptotic signaling in 4910 cancer stem cells and U87 glioma cells. Following treatment, an increase in the cleaved 35 kDa fragment of Caspase-9 was observed, which was indicative of Caspase-9 activation. This then triggered pro-apoptotic pathways following the effector caspase cleavage and activation. The overexpression of miR-211 also inhibits glioma cell invasion and migration via suppressing *MMP9* expression. Other known targets of miRNAs in cancers include *MMP2*, *MMP9*, *MMP13*, *MMP14*, and *MMP16* and their substrates such as type I, II, and IV collagens [22,23].

### 3.2. Cardiovascular Diseases

Aberrant activity of MMPs contributes to the development and progression of atherosclerosis by influencing the local inflammatory response at the site of atherosclerosis plaque formation and by catalyzing plaque rupture, an event that ultimately culminates in myocardial infarction and heart failure [44]. Recent evidence implicates epigenetic control of MMP expression in multiple cardiovascular pathologies, including atherosclerosis. The induced hypomethylation of *MMP2* and *MMP9* via microRNA-29b upregulation is a mechanism by which oxidized low-density lipoprotein (oxLDL) contributes to atherosclerosis [45]. The oxLDL promotes atherosclerotic plaque formation by inducing smooth muscle cell migration and proliferation. In human aortic SMC, oxLDL induced cell migration via the upregulated expression and activation of MMP-2 and MMP-9 due to the fact that both MMPs promote smooth muscle cell migration [46]. Analysis of the DNA methylation status of *MMP2* and *MMP9* following oxLDL treatment revealed significant demethylation in the two genes [45]. DNMT3b mRNA and protein levels in human aortic SMCs significantly decreased after oxLDL treatment [45]. DNMT3b knockdown in oxLDL-treated human aortic SMCs significantly increased *MMP2* and *MMP9* mRNA levels, indicating that *MMP2* and *MMP9* were repressed by DNA methylation. These results show that upregulated *MMP2* and *MMP9* expression resulted from decreased DNA methylation due to DNMT3b downregulation. Further, Chen et al. (2011) also determined that microRNA-29b was upregulated in a dose-and time-dependent manner in oxLDL treated SMCs [45]. These results suggest that microRNA-29b indirectly modulates the DNA methylation of *MMP2* and *MMP9*, and thus their expression, by regulating the expression of DNMT3b.

Thus, DNA methylation leading to aberrant expression of MMPs can significantly influence the progression and development of cardiovascular diseases. Similarly, the hypomethylation of the *MMP9* gene and the aberrant upregulation of *MMP9* expression is observed in Kawasaki disease, a pediatric vascular disease characterized by inflammation of the coronary arteries [47]. The methylation status of the CpG sites of *MMP9* shows a strong negative correlation with mRNA expression levels, indicating that *MMP9* is repressed by DNA methylation [47]. Furthermore, a differential methylation status in all MMP genes was induced by intravenous immunoglobulin treatment in Kawasaki disease patients [47] (Figure 1).

### 3.3. Bone/Cartilage Diseases

MMPs modulate bone growth, development, and repair processes through their actions on extracellular matrix components, and dysregulated MMP expression and activity are thus implicated in bone diseases such as osteoarthritis (OA) and rheumatoid arthritis [48] (Figure 1). A genome-wide analysis of DNA methylation status involving osteoarthritis patients found the *MMP3*, *MMP9,* and *MMP13* genes to be hypomethylated (with increased gene expression) in osteoarthritis versus non-osteoarthritis chondrocytes [49,50]. The percentage of methylated CpG sites of these MMP genes in clonal chondrocytes from osteoarthritis patients versus controls were: 80% versus 96% for *MMP13*, 19% versus 53% for *MMP9*, and 43% versus 70% for *MMP3* [50]. The study concluded that the altered expression of cartilage-degrading MMPs resulted from clonally heritable epigenetic changes that may exacerbate osteoarthritis.

MicroRNA-222 and microRNA-27b regulate *MMP13* expression in osteoarthritis chondrocytes [51]. Song et al. (2015) found that miR-222 expression is downregulated and its target HDAC-4 is consequently upregulated in OA chondrocytes [51]. Elevated HDAC-4 expression thereby increased *MMP13* expression via histone de-acetylation. The study also showed that the inhibition of HDACs by Trichostatin A (TSA) lowered the HDAC-4 and MMP-13 protein expression in OA chondrocytes, thereby confirming the role of HDAC-4 in regulating MMP-13. When overexpressed, MMP-13 can exacerbate OA [52]. Akhtar et al. (2010) found a direct inhibitory effect of miR-27b on the expression of the *MMP13* gene in the OA chondrocytes [52]. MicroRNA miR-27b interacts with the 3′-untranslated region of *MMP13* mRNA, which contains a complementary sequence to the miR-27b seed sequence and downregulates *MMP13* expression at the post-transcriptional level [52]. MiR-27b expression was found to be significantly reduced in OA cartilage when compared to normal cartilage. OA chondrocytes exhibiting higher levels of miR-27b produced significantly less MMP-13 protein upon IL-1*β* stimulation compared to chondrocytes that were not transfected with a miR-27b mimic, and inhibition of miR-27b increased MMP-13 protein expression in IL-1*β*–stimulated chondrocytes when compared to control OA chondrocytes. These findings indicate that miR-27b acts as a regulator of MMP-13 protein expression in human OA chondrocytes [52].

In rheumatoid arthritis (RA), abnormal histone modifications and DNA methylation both lead to aberrant upregulation of MMP gene transcription [53] (Figure 1). Genome-wide DNA hypomethylation is observed in rheumatoid arthritis synovial fibroblasts (RASF), resulting in the upregulated expression of 186 genes including *MMP1* and *MMP14* [54]. In RASF, histone H3 lysine-4-trimethylation (the histone marker for active transcription) is increased, while transcription-repressive H3 lysine-27-trimethylation is decreased in the promoters of *MMP1*, *MMP3*, *MMP9,* and *MMP13* [55]. Interestingly, despite increased active histone markers in its promoter, *MMP9* expression was not upregulated by interleukin-6 (IL-6) induction, while *MMP1*, *MMP3,* and *MMP13* were upregulated. This occurs as a result of the IL-6-inducible transcription factor STAT3 being recruited to the promoters of *MMP1*, *MMP3*, and *MMP13*, but not to the *MMP9* promoter [55]. The differential expression of MMP genes with similar histone methylation states suggests that epigenetic mechanisms work in concert with the canonical mechanisms of MMP transcription control.

Furthermore, recent evidence suggests that *MMP9* expression in RA may also be epigenetically regulated by miRNAs. Wang et al. (2019) reported that the fibroblast-like synoviocytes from patients with RA exhibited elevated miR-145-5p expression and increased *MMP9* mRNA and protein levels [56]. The overexpression of miR-145-5p induced the nuclear translocation of p65, thereby activating NF-κB which induced the expression of *MMP9* by binding the *MMP9* promoter region [56]. These demonstrate that miR-145-5p can indirectly modulate the expression of *MMP9* in patients with RA [56].

### 3.4. Bacterial Infections—Tuberculosis

Epigenetic regulation of MMP-1 and MMP-3 (a physiological MMP-1 activator) modulates the immune response against *Mycobacterium tuberculosis* (Mtb) in pulmonary tuberculosis [57,58,59,60]. In a study by Moores et al. (2017), Mtb infection decreased gene expression of class I histone deacetylases (HDACs 1, 2, 3, and 8) by more than 55% in human Mtb-infected macrophages [57]. No significant decrease in the gene expression of class I HDACs was seen in normal human bronchial epithelial (NHBE) cells stimulated with conditioned medium from Mtb-infected monocytes (CoMTb) [57]. The inhibition of HDACs (with trichostatin A and m-carboxycinnamic acid bis-hydroxamide) reduced MMP-1 and MMP-3 secretion in Mtb-infected macrophages and CoMTb-stimulated NHBEs [57]. However, the HDAC class I-specific inhibitor, MS-275, increased MMP-1 and MMP-3 secretion in CoMTb-stimulated NHBEs while decreasing MMP-1/-3 secretion in Mtb-infected macrophages [57]. Therefore, altered histone acetylation in Mtb infection may affect MMP-1/-3 expression. The study also showed that inhibition of histone acetyltransferases (HATs), using HATi II, lowered MMP-1 expression and secretion by more than 50% as well as significantly lower MMP-3 secretion in Mtb-infected macrophages [57]. HAT inhibition was also confirmed using another HAT inhibitor, anacardic acid, which significantly lowered MMP-1 and MMP-3 secretion in Mtb-infected macrophages [57]. These results indicate that acetylation by HATs is needed for MMP-1 and MMP-3 expression and secretion in Mtb-infected cells. Moreover, an increased histone H3 and H4 acetylation of the *MMP1* promoter region was reported in CoMTb-stimulated NHBEs compared to the uninfected controls. This accompanied a more-than-10 fold increase in RNA polymerase II binding to the *MMP1* promoter [57]. These results suggest that the epigenetic modification in the promoter region induced by Mtb infection can alter *MMP1* gene expression.

The effects of HDACs and HATs on MMP expression can be more complex and indirect, due to interrelated signaling cascades involving transcription factors and non-histone proteins that can also be substrates of the above-mentioned enzymes [61,62,63]. Such non-histone substrates include HDACs themselves [63], in addition to the NF-κB and AP-1 family of transcription factors, which are believed to regulate *MMP1* gene expression [60,64]. To further demonstrate the role of non-histone proteins in mediating the regulation of HDAC on MMP expression, Liu et al. (2003) reported that upregulation of the reversion-inducing-cysteine-rich protein with kazal motifs (RECK), a reported tumor invasion and angiogenesis suppressor [65], induced by HDAC inhibition downregulates the activation of MMP-2 [66]. The HDAC inhibitor trichostatin A upregulated RECK gene expression in CL-1 human lung cancer cells [66]. Trichostatin A-treated CL-1 cells exhibited inhibition of MMP-2 activity. This was not caused by the downregulation of *MMP2* gene expression, but rather the downstream effects of trichostatin A-induced RECK upregulation, which decreased CL-1 cancer cell invasion [66]. The results were confirmed using siRNA to suppress RECK expression, which restored the trichostatin A-induced inhibition of MMP-2 activity [66]. Barchowsky et al. (2000) reported the involvement of the inflammatory cytokine IL-1 in the activation of *MMP1* gene expression in rabbit primary synovial fibroblasts (RSF) [64]. The activation of MMP transcription by IL-1 is mediated through the NF-κB pathway [64]. In addition to IL-1, TNF-α can also induce gene expression of MMPs, such as the *MMP1* and *MMP13* genes, via downstream phosphorylation pathways [67,68]. These pathways include the NF-κB and the mitogen-activated protein kinase (MAPK) pathways, and the MAPK pathway can, in turn, activate downstream transcription factors such as AP-1 and induce MMP expression along with NF-κB in a parallel pathway [67,68]. Interestingly, the network between the mediators of signaling pathways and epigenetic changes has revealed an interaction between HDAC-1 and the NF-κB subunit p65 (RelA) that in turn suppresses the expression of NF-κB-regulated genes [69], which can include MMPs. Therefore, the involvement of the phosphorylation pathways and the subsequent involvement of transcription factors may explain the indirect effect of HDAC inhibition on *MMP1* expression, as reported in tuberculosis and the cytokine-induced expression of MMP-1/-13 proteins in human articular chondrocytes. The relationship between HDAC/HATs activity and MMP expression and activity is influenced by determinants of inflammatory status such as the levels of pro-inflammatory cytokines (e.g., IL-1 and TNF-α) and transcription factors (e.g., AP-1 and NF-κB).

To further delve into examples of cytokine-mediated intermediary signaling pathways, Mittelstadt and Patel (2012) reported the inhibitory effect of the anti-proliferative cytokine interferon-β (IFN-β), on *MMP9* gene expression in human HT1080 fibrosarcoma cells [70]. IFN-β decreased NF-κB recruitment, however, it increased HDAC-1 recruitment to the promoter-proximal AP-1 site of *MMP9*, subsequently reducing histone H3 acetylation [70]. IFN-β-induced post-translational modifications of the transcription factor AP-1 repolarized its role from being pro-transcription to anti-transcription, which may be facilitated by its increased binding to and recruitment of HDAC-1 [70]. Therefore, such inhibition of *MMP9* expression could reduce its pro-angiongenic, inflammatory, and metastatic roles (Figure 1), and thus alter its extensive involvement in infections and auto-immune diseases such as arthritis, lupus, and multiple sclerosis [70,71,72].

MMPs can contribute to infection-induced endotoxic shock through their altered expression in the disease state, in which they modulate the secretion of inflammatory cytokines and degrade the proteins of the endothelial basement membrane, such as type IV collagen [73]. Such cleavage of the basement membrane proteins facilitates the dissemination of pathogens to distant organs and results in systemic endotoxic shock [73]. The expression of several MMPs in macrophages, including MMP-1, -7, and -9, can be induced in infection states via inflammatory stimuli such as bacterial lipopolysaccharides (LPS) [74]. In bone marrow-derived macrophages (BMM), bacterial LPS induced the expression of the HDACs 1, 4, 5, and 7 [75]. In addition, LPS induced HDAC-2 expression in macrophages, accompanied by a pro-inflammatory immune response [75]. Therefore, the expression of MMPs, such as MMP-1 and/or MMP-9, can be epigenetically regulated in multiple diseases influenced by HDACs, as mentioned previously, including inflammatory conditions such as rheumatoid arthritis, bacterial infections, and cancer metastasis. Considering the overall interplay between the phosphorylation signaling pathways, inflammatory cytokines, and mediators of epigenetic changes, the expression of MMPs can be altered indirectly, on both the genetic and epigenetic levels, consequently exacerbating the pathogenesis of these diseases.

### 3.5. Viral Infections

Multiple viruses including the hepatitis C virus (HCV), the middle east respiratory syndrome coronavirus (MERS-CoV), and H5N1 avian influenza (HPAI) impact the epigenetic regulatory mechanisms in the genome of the host cell [76,77,78]. HCV via its core protein can interfere with H_2_O_2_ (a reactive oxygen species)-induced apoptosis in human hepatocytes through the p53-dependent pathway by epigenetically inhibiting its modulator p14 [76]. The core protein of HCV inhibits p14 expression via inducing hypermethylation of its promoter region, consequently downregulating p53 levels [76]. Coronaviruses, such as MERS-CoV, and influenza viruses, such as HPAI, downregulate the antiviral interferon (IFN)-stimulated genes by inducing inhibitory histone methylations [77]. Furthermore, MERS-CoV, through DNA methylation, and H5N1-VN1203, through both DNA methylation and histone modifications, antagonize host gene expression of IFN-γ-related antigen-presentation-associated genes [78]. On the other hand, H1N1-09 and severe acute respiratory syndrome coronavirus (SARS-CoV) infections resulted mostly in the strong upregulation of IFN-γ–responsive genes [78].

Likewise, the highly infective severe acute respiratory syndrome coronavirus 2 (SARS-CoV-2), the causative agent of coronavirus disease-19 (COVID-19), also impacts the host’s epigenome environment [79]. The entry of the SARS-CoV-2 into the host’s cell relies on how the viral spike (S) glycoprotein interacts with the host cell receptor, angiotensin-converting enzyme 2 (ACE2) receptor, accompanied by S protein priming mediated by the cellular serine protease TMPRSS2, which cleaves the S protein [80]. SARS-CoV-2 infection induces oxidative stress, which aggravates epigenetic changes such as the DNA hypomethylation of the *ACE2* gene, leading to ACE2 overexpression, which may further increase the susceptibility of host cells to SARS-CoV-2 infection [81]. In a recent report, SARS-CoV-2 infection resulting in severe COVID-19 was associated with changes in genome-wide DNA methylation in peripheral blood mononuclear cells, and comparing severe COVID-19 versus uninfected controls identified 40,904 differentially methylated loci, whereas COVID-19 versus influenza identified 26,733 differentially methylation loci [82]. In severe COVID-19 cases, the gene of the transcription factor STAT3, which binds to the promoters of *MMP1*, *MMP3*, and *MMP13* [55] and regulates their expression, had eight hypermethylated CpG sites, which indicates an epigenetic inhibition of STAT3 expression [82]. Circulating MMP-9 is elevated in severe COVID-19, although the mechanism of this upregulation is unclear [83]. These data show that viral infections such as SARS-CoV-2 can induce genome-wide epigenetic changes in host cells, and could thus potentially affect the expression of the MMPs that play important roles in the pathogenesis of acute respiratory distress syndrome through the degradation and remodeling of the lung extracellular matrix [82,84,85].

## 4. Pharmacologically Induced Epigenetic Control of MMPs

### 4.1. Epigenetic-Based Therapeutic Control of MMPs and Mediators of Epigenetic Changes

Elucidating the epigenetic mechanisms involved in chronic diseases and infections can be critical not only to understand the pathogenesis mechanisms of these diseases but also to design novel therapeutic interventions. For instance, in tuberculosis, the inhibition of HDACs using suberanilohydroxamic acid (SAHA), also known as the drug Vorinostat, resulted in an immune response by macrophages and subsequently T cells against Mtb [86]. This immune response involved increased production of IL-1β and reduction of IL-10, accompanied by stimulation of T cells in a human Mtb-infected macrophage culture system [86]. Furthermore, in water-borne parasitic infections by *Cryptosporidium* species (causing cryptosporidiosis) such as *C. parvum,* Vorinostat showed anti-parasitic in vitro and in vivo potential by targeting the HDAC of *C. parvum* [87]. Furthermore, in human articular chondrocytes, fibroblast growth factor-2 along with IL-1β-induced expression of MMP-1/-13 can lead to cartilage degradation [88]. However, upon HDAC inhibition using trichostatin A (TSA), the induced expression of these MMPs was decreased [88]. Therefore, our understanding of these inflammatory pathways and their relationships to the expression of MMPs is essential in therapeutic designs based on the epigenetic-mediated inhibition of MMP expression. For instance, Young et al. (2005) reported the favorable effects of the HDAC inhibitors trichostatin A and sodium butyrate in reducing cartilage degradation mediated by the pro-inflammatory cytokines-induced expression of MMP-1/-13 [30].

The MMP-2-mediated degradation of the extracellular matrix (ECM) in periodontitis can be epigenetically induced by the spirochete *T. denticola* [89]. During the *T. denticola* challenge of periodontal ligament (PDL) cells, the induced expression of the *MMP2* gene was reversed with HDAC inhibition treatment using apicidin and trichostatin A, in addition to inhibitors of histone phosphorylases and DNA methyltransferases [89]. Thus, treatment strategies involving HDAC-inhibiting drugs are successful candidates for managing rheumatoid arthritis and possibly bacterial infections, which highlights the importance of investigating the inhibition of other mediators of epigenetic modifications affecting MMP expression in infections and other diseases.

### 4.2. Statins

MMP gene expression is altered at the transcriptional and post-transcriptional levels by statins [16,17], a family of commonly prescribed drugs for lowering plasma low-density lipoprotein cholesterol levels [90]. Statins induce epigenetic changes on the genes involved in atherosclerosis development, cell-cycle progression, and tumorigenesis [18,91,92,93,94]. Increased histone acetylation through the inhibition of HDACs is an anti-cancer therapeutic strategy [95,96]. Atorvastatin, fluvastatin, lovastatin, pravastatin, and simvastatin inhibited HDAC activity and increased acetylation levels of histone H3 in human A549 lung carcinoma cells [94]. These statins increased the mRNA expression of p21, a cyclin-dependent kinase inhibitor, by inducing HDAC 1 and 2 removal and allowing for increased histone H3 acetylation at the *p21* promoter [94]. The increased p21 expression resulted in inhibition of tumor proliferation [94]. DNA hypermethylation and inactivation of tumor suppressor genes is a therapeutic target in tumor formation [97]. Karlic et al., (2015) reported the downstream epigenetic effects of statins, in which simvastatin induced downregulation of DNA methyltransferases in human cancer cell lines including ovarian cancer, breast cancer, prostate cancer, and osteosarcoma [92]. Furthermore, simvastatin induced the downregulation of HDAC-1, -2, -3, -7, and -8 mRNA expression [92]. Thus, statins exert downstream anti-cancer potential through epigenetic modifications [91,92,94] mediated by the common epigenetic regulators of MMPs.

The epigenetic effects of rosuvastatin, atorvastatin, and lovastatin are involved in atherosclerosis regression, where they induce the mRNA expression of the chemokine receptor CCR7 in mice RAW264.7 macrophages [93]. Rosuvastatin induces CCR7 expression partly by releasing class II HDACs from the *CCR7* promoter region and increasing histone H3 and H4 acetylation to the same region [93]. Consequently, rosuvastatin induces plaque regression through the removal of macrophages from atherosclerotic plaque, which is mediated by CCR7 activation [93].

Atorvastatin, Fluvastatin, and simvastatin show impacts on MMP gene expression [17,98,99]. They inhibited MMP-1 and MMP-3 secretion from naïve and cytokine-stimulated human fetal lung fibroblasts [98].

Treatment of human vascular endothelial cells with fluvastatin and lovastatin inhibited the expression of MMP-1, but not the expression of the tissue inhibitor of MMP-1 [99]. Fluvastatin reduced MMP-9 secretion and activity from mouse macrophages [100]. Lovastatin and cerivastatin inhibited the secretion of MMP-1, -2, -3, and -9 in human vascular smooth muscle cells [17]. Simvastatin inhibited MMP-9 expression in rat alveolar macrophages exposed to cigarette smoke extract [101]. Conceivably, long-term treatment with statins is likely to alter the transcriptional control of many MMP genes involved in atherosclerosis, a hypothesis that warrants investigation.

### 4.3. Tetracyclines with MMP Inhibitory Actions

Tetracyclines exert antibiotic effects by binding to bacterial 30S ribosomal subunits to inhibit bacterial protein synthesis [19]. In addition to their antimicrobial use, some tetracyclines (e.g., minocycline and doxycycline) exhibit MMP-inhibitory properties [102,103,104,105] through their ability to: (i) bind metal ions required for the catalytic activity of MMPs [102], and (ii) inhibit MMP gene transcription [103,104,105]. Doxycycline, the tetracycline with the strongest MMP inhibitory actions [102], has been approved as an MMP inhibitor by various regulatory bodies, including Health Canada and the U.S. FDA, for the treatment of periodontitis and rosacea. No other MMP inhibitor has received FDA approval for treating human disorders, largely because, in early trials, various MMP inhibitor structures caused major adverse effects including increased mortality in cancer patients [106]. Interestingly, in the recent TIPTOP trial [107], low-dose doxycycline (100 mg b.i.d. for 7 days) reduced left-ventricular hypertrophy and dysfunction in patients with acute and severe myocardial infarction. Based on these promising results, two new trials were approved to explore the application of doxycycline in myocardial infarct patients in Australia (ACTRN12618000467235) and Canada (ClinicalTrials.gov Identifier: NCT03508232). Whether MMP inhibition or the epigenetic mechanisms leading to decreased MMP activity underlie the benefits of doxycycline in these cardiac infarct patients remains unclear. However, emerging evidence points at possible epigenetic mechanisms of doxycycline that merit consideration from a therapeutic point of view. For instance, in models of abdominal aortic aneurysm, a vascular disease associated with the overexpression of MMP-2 [104], doxycycline concentration-dependently inhibited the secretion of MMP-2 in cultured human smooth muscle cells and abdominal aortic aneurysm explants [104], which was accompanied by a reduction in the half-life of *MMP2* mRNA from 49 h to 28 h [104]. A converging line of research indicates that the doxycycline inhibition of MMP-2 expression may involve post-transcriptional mRNA destabilization and downregulation of *MMP2* gene expression in cultured human skin keratinocytes [105]. Further, doxycycline can also alter microRNA expression [108]. Regulatory T cells in mice treated with doxycycline showed increased expression of the miR-486 family of microRNA (miR-486-3p and miR-486-5p) after a four-week recovery period following doxycycline treatment [108] with recent studies showing that the *MMP2* and *MMP9* mRNAs are targets of miR-486 [109,110,111]. In particular, induction of miR-486-5p suppressed MMP-2 and MMP-9 protein expression in human colorectal cancer cell lines [110] as well as in human lung adenocarcinoma cell lines [111], suggesting that an epigenetic mechanism by which doxycycline affects MMP expression occurs via the induction of miR-486. Doxycycline can indirectly impact MMP expression by altering microRNA/mRNA expression.

## 5. Conclusions

Various epigenetic mechanisms: DNA methylation, histone acetylation/methylation, and alterations in small non-coding RNAs can influence the expression of MMPs, tissue inhibitors of MMPs, and the transcription factors that regulate the expression of MMPs (Table 1). Highly prevalent diseases and commonly prescribed medicinal drugs can introduce epigenetic pressure through these mechanisms to alter the proteolytic activity of MMPs. Bacterial and viral infections also induce epigenetic pressure to directly or indirectly affect the expression of MMP genes. Interestingly, SARS-CoV2 infection induces genome-wide DNA methylation, affecting a multitude of genes, including MMP transcription factors, and is likely to directly or indirectly change the MMP gene expression of profile in infected host cells. Our understanding of (and our ability to effectively treat) human conditions associated with alterations in the proteolytic activity of MMPs must consider that these conditions and their medicinal treatments are potential causes of epigenetics-related alterations in MMP activity. Whether the epigenetic mechanisms affecting the activity of MMPs can be therapeutically targeted is unclear and warrants further research. Future research should have the goal of understanding and confirming the effects of the short- and long-term epigenetic pressures induced by MMP-associated pathologies and medicinal drugs on the expression of MMPs, endogenous MMP inhibitors, and MMP substrates. Although the effects of statins on the expression of MMPs are well documented, more research is needed to establish the involvement of specific epigenetic mechanisms. How epigenetic pressure induced by viral and bacterial infections affects MMP expression warrants determination through future research. The regulation of gene expression by miRNAs might open opportunities for the selective manipulation of MMP expression and signaling when pharmacological inhibitors are not available. The future of MMP biology appears to be as exciting and rewarding as ever.

## Figures and Tables

**Table 1:** Examples of epigenetic mechanisms that regulate gene expression of MMPs and TIMPs.

Target Gene	Epigenetic Control Mechanism	Reference
*MMP1*	▪ DNA methylation ▪ histone acetylation and chromatin remodeling ▪ H3 lysine-27-trimethylation	[23,54] [29,30,57] [55]
*MMP2*	▪ DNA methylation of CpG sites and histone H3 lysine-27-trimethylation ▪ Targeted by miR-488, miR-206, miR-29b, miR-451, miR-486-5p ▪ *GRK6* promoter hypermethylation	[35,36] [22,45,109,110] [41]
*MMP3*	▪ DNA methylation by DNA methyltransferases (e.g., *Dmt-1* and *Dmt3b*) ▪ H3 lysine-27-trimethylation ▪ histone acetylation and chromatin remodeling	[25,49,50] [55] [57]
*MMP7*	▪ GRK6 promoter hypermethylation	[41]
*MMP9*	▪ DNA methylation at the *MMP9* promoter ▪ H3/H4 acetylation ▪ miR-206, miR-211, miR-218, miR-212/132, miR-29b, miR-451, miR-486-5p ▪ H3K4me2 and H3K4me3 methylation by SMYD3 ▪ H3 lysine-27-trimethylation	[24,47,49,50] [28] [22,43,110,111] [37,42] [55]
*MMP10*	▪ DNA methylation of promoter ▪ histone acetylation and chromatin remodeling	[25] [29,30]
*MMP13*	▪ DNA methylation ▪ miR-143, miR-27b, miR-222 ▪ H3 lysine-27-trimethylation ▪ histone acetylation/de-acetylation and chromatin remodeling	[49,50] [22,51,52] [55] [29,30,51]
*MMP14*	▪ DNA methylation of CpG sites and histone H3 lysine-27-trimethylation ▪ miR-10b	[35,36,54] [22]
*MMP16*	▪ miR-146b	[22]
*TIMP2*	▪ DNA methylation of two CpG sites in the promoter	[35]
*TIMP3*	▪ miR-181b, miR-221/222	[22]

## Data Availability

Not applicable.
